# miR-205 Expression Elevated With EDS Treatment and Induced Leydig Cell Apoptosis by Targeting RAP2B via the PI3K/AKT Signaling Pathway

**DOI:** 10.3389/fcell.2020.00448

**Published:** 2020-06-09

**Authors:** Yang Cui, Rui Chen, Lin Ma, Wenjing Yang, Mingyue Chen, Yanghai Zhang, Shuai Yu, Wuzi Dong, Wenxian Zeng, Xianyong Lan, Chuanying Pan

**Affiliations:** ^1^Key Laboratory of Animal Genetics, Breeding and Reproduction of Shaanxi Province, College of Animal Science and Technology, Northwest A&F University, Xianyang, China; ^2^Key Laboratory of Animal Biotechnology, Ministry of Agriculture, Northwest A&F University, Xianyang, China

**Keywords:** pig, Leydig cell, mRNA transcripts, miR-205, apoptosis

## Abstract

The adult Leydig cells (ALCs), originated from stem Leydig cells (SLCs), can secrete testosterone which is essential for germ cell development and sexual behavior maintenance. As a synthetic compound, ethane dimethane sulfonate (EDS), a well-known alkylating agent, has been reported to specifically ablate ALCs. In this study, EDS was verified to ablate differentiated pig LCs by experiments. Subsequently, the primary isolated pig LCs (containing SLCs and differentiated LCs) and EDS-treated LCs (almost exclusively SLCs) were collected for RNA-seq 4,904 genes and 15 miRNAs were differently expressed between the two groups. Down-regulated genes in the EDS-treated group were mainly related to steroid hormone biosynthesis. The highest up-regulation miRNAs was miR-205 after EDS treatment. Additionally, miR-205 was expressed more highly in pig SLCs clones compared with differentiated LCs. Through qRT-PCR, western blot (WB), TUNEL, EDU and flow cytometry, miR-205 was found to induce cell apoptosis, but did not affect proliferation or differentiation in both TM3 and GC-1spg mouse cell lines. Through luciferase reporter assays and WB, RAP2B was identified as a target gene of miR-205. Besides, overexpression of miR-205 inhibited the expressions of PI3K, Akt and p-AKT. All these findings were helpful for elucidating the regulation mechanism in pig LCs.

## Introduction

The globally increasing male reproductive system abnormality is getting more and more attention. Evidences come from a meta-analysis confirmed the sperm concentration and male fertility declining in the past two decades (Rapoport, 1988). Male reproductive ability decreasing is more likely to be related to environmental factors rather than genetics (Le Moal et al., 2014). Recently, several studies pointed out an association between synthetic chemicals exposure and male reproductive disorders (Albert et al., 2018; Huang et al., 2019). As a synthetic compound, EDS, a well-known alkylating agent, has been reported to specifically ablate ALCs (Ge et al., 2006). Importantly, in mammals, the main function of ALCs is T synthesis and secretion, which not only plays an essential role in maintenance of somatic cell function and germ cell development, but also maintains male phenotype and sexual behaviors (Smith and Walker, 2014). These function imply that EDS would cause serious damage of male reproduction. However, the mechanism of EDS ablating Leydig cell remains unclear.

Leydig cell is the major cell type present in the interstitial compartment of testis. In rats, it has been demonstrated that ALCs originate from undifferentiated mesenchymal-like stem cells, called SLCs (Ye et al., 2017). SLCs are spindle-shaped cells and express PDGFRα, LIF receptor (LIFR), Thy-1, Nestin, as well as some other stem cell markers (Ge et al., 2006; Inoue et al., 2018). The postnatal development process of rat LCs can be artificially divided into three distinct stages: PLCs arising around postnatal day 21 (PND21) (Ye et al., 2017), ILCs around postnatal day 28 (Ge and Hardy, 1998) and most of ILCs eventually mature into ALCs by day 56 (Chen H. et al., 2009). To date, the *in vitro* culture system of rat LCs has been established (Klinefelter et al., 1987; Wang et al., 2019), and the existence of LCs was also confirmed in human and mice (Lo et al., 2004; Gao et al., 2018). What is noticeable is that few studies about LCs have been carried out in other mammalian animals, except those mentioned above.

Pig is an important animal model for human disease studies because of its high similarity with human physiological characteristics and genome size (Bergfelder-Druing et al., 2015). In 2017, our research group, for the first time, effectively established the *in vitro* short-term culture system for pig SLCs (Yu et al., 2017). Our study found that the PDGFRα positive spindle-shaped cells existed in the peritubular regions of 7-day-old pig testes. Then, the primary pig LCs were obtained by enzymes digesting method and subsequently cultured with DMEM-F12 plus testicular fluid from a piglet (called pTF medium). Theoretically, the 7-day-old pig testes not only contained SLCs, but also included other differentiated LCs. Through the immunofluorescent analysis of cytochrome P450 family 17 subfamily A polypeptide 1 (CYP17A1), differentiated pig LCs could be specifically eliminated by EDS (Yu et al., 2017). However, the genes or non-coding RNAs that participate in the regulation of pig SLCs proliferation and differentiation remain unknown.

Currently, almost none studies focus on the mechanism of mRNAs or miRNAs participate in the regulation of EDS ablating pig LCs. In this study, high-throughput sequencing was performed on newly isolated pig primary LCs (containing SLCs and differentiated LCs) and EDS-treated LCs (primary cell types were SLCs). Compared with the primary group, EDS treatment group had 2,249 genes up-regulated and 2,645 genes down-regulated. GO annotation and KEGG analysis found that EDS treatment group significantly down-regulated gene targeting “steroidal biosynthesis” pathway. Additionally, 15 known miRNAs were significantly differentially expressed between primary group and the EDS-treated group, with miR-205 being the most up-regulated miRNA in the EDS-treated group. Subsequently, the expression of miR-205 was verified on pig SLCs clones and differentiated LCs. The results showed that miR-205 was highly expressed in porcine SLCs clones, so miR-205 was selected for further study. miR-205 was verified to have no effect on proliferation of TM3 cells and GC-1spg cells, but it induce apoptosis by targeting RAP2B gene by multiple experimental methods. Meanwhile, miR-205 inhibits PI3K/Akt pathway. The mRNAs and miRNAs database, especially the DEGs and miRNAs information can provide a basis for further understanding of pig LCs.

## Materials and Methods

### Animal and Tissue Samples Collection

Animal experiments in this study were approved by the Faculty Animal Policy and Welfare Committee of Northwest A&F University (protocol number NWAFAC1008). The care and use of experimental animals fully complied with local animal welfare laws, guidelines, and policies.

The 7-day-old male pig fresh testis samples were obtained from Besun Agricultural Industry Group Co., Ltd. (Yangling, Shaanxi, China). The samples were quickly placed in DPBS which containing penicillin-streptomycin (P/S) solution (Invitrogen, Carlsbad, CA, United States) and returned to the laboratory for following experiments within 1 h (Yu et al., 2017). SSCs and SCs were separated using the method of Zhang P. et al. (2017). For expression patterns experiment, 11 tissues of four Guanzhong black pigs were harvested, including heart, liver, spleen, lung, kidney, large intestine, small intestine, brain, muscle, testis, and epididymis.

### Hematoxylin and Eosin (H&E) Staining and Immunohistochemical Analysis

Testis samples of 7-day-old pigs were fixed in Bouin’s solution for 18 h. Then the tissues were dehydrated and embedded in paraffin. After the paraffin solidified, it was cut into 5 μm and adsorbed on a glass slide. Finally, the Hematoxylin and Eosin (H&E) staining was used to observe histology (Heidari et al., 2012).

For immunohistochemistry, the protocols were performed as previously described (Yu et al., 2017). In brief, the paraffin sections were deparaffinized, rehydrated, and rinsed in PBS. Antigen retrieval used boiling method in a solution of 0.01 M Tris-EDTA (pH = 9.0) for 10 min, followed by incubation with 10% donkey serum for 2 h at 37°C. Primary antibodies (anti-PDGFRα, 1:200, Abcam, Cambridge, United Kingdom) incubated overnight at 4°C. Secondary biotinylated antibodies (ZSGB-BIO, China) incubated for 1 h at 37°C. Finally, 3,3’-diaminobenzidine (DAB, ComWin Biotech, China) was used to detect protein expression. Digital images were captured with a microscope camera (Tokyo, Japan).

### Isolation of Pig LCs

The pig LCs was isolated following the procedure as described by Yu et al. (2017). Briefly, the testes were minced. Ml collagenase type IV (Invitrogen) was used to disperse cells in tissues. The target cells were separated by filtration and differential centrifugation. Subsequently, the erythrocyte in the cell suspension was removed using hyaluronidase (Invitrogen) and blood cell lysate. Finally, differential plating was used to obtain LCs (Ohno et al., 2005). About half of the cells were stored using Trizol reagent (TaKaRa, Dalian, China), and the other were cultured in DMEM/F12 medium with EDS treatment. Six separate experiments were conducted. Three samples of each treatment were used for RNA sequencing sample collection, and the other three samples were used for experimental validations.

### Ethane Dimethane Sulfonate (EDS) Treatment

Previous studies reported that EDS could selectively ablate all the differentiated LCs in the rat testis (Sharpe et al., 1990; Morris et al., 1997; Wu et al., 2017). The blank control (not contain DMSO), 0 mg/mL EDS, and 1.0 mg/mL EDS was added to the culture solution (Yu et al., 2017). After treatment for 24 h, the cells were collected using Trizol reagent (TaKaRa, Dalian, China) and stored at −80°C for RNA extraction. Six separate experiments were conducted.

### RNA Extraction, Libraries Generation and Sequencing

After treatment for 24 h, the cells were collected using Trizol reagent (TaKaRa, Dalian, China) and stored at −80°C for RNA extraction according to the method previously described (Chen R. et al., 2017).

Novogene (Beijing, China) detected the purity and concentration of RNA samples, constructed libraries, and sequenced using the Illumina HiSeq 2000 (LianChuan Sciences, Hangzhou, China) sequencing platform. Note that, we eliminated two low quality samples. Finally, we obtained eight raw sequencing files of the transcriptome of pig LCs, four mRNA fastq files and four miRNA fasta files. Among them, four samples from the original generation of digestion, named the primary group, and four samples with 1.0 mg/mL EDS treatment for 24 h, named the EDS-treated group, by deep RNA sequencing method.

### Analysis of Sequencing Data

FastQC (version 0.10.1) evaluated the quality of the raw sequencing reads, and Trimmomatic (version 0.38) filtered low-quality reads and reads containing adapter to get clean reads for further analysis (Bolger et al., 2014). For mRNA-seq, the TopHat (version 2.1.0) was used to map clean reads to the pig genome (Sscofa 10.2.88) (Trapnell et al., 2009). Next, the reads count of 21,607 protein-coding gene in pig were estimated by HTSeq (version 0.6.0) (Quinlan and Hall, 2010) using the default parameters. For miRNA-seq, we only retained reads with a length ranging from 18 to 30 nt based on miRNA characteristics, and the reads were mapped to the pig genome by mapper. pl script of miRDeep2 software (version 0.1.0). Meanwhile, the miRDeep2. pl script calculated the reads count of known pig miRNA sequence that derived from the miRBase website (Friedlander et al., 2012). All of the raw sequence data were submitted to the NCBI SRA database: SRR11625159, SRR11625160, SRR11625161, SRR11625162, SRR11625155, SRR11625156, SRR11625157, and SRR11625158.

### Differential Expression Gene or miRNA Analysis and Gene Functions Analysis

Based on the reads count of each sample, this study screened the differential expression genes (DEGs) or miRNAs between the two treatment groups using R package edgeR (Robinson et al., 2010). Corrected *P*-value < 0.05 and the log2FC (fold-change) > 2 were used to define DEGs. GO^1^ was used to annotate the function of DEGs. KEGG pathway analysis were performed using DAVID (version 6.8^2^). The corrected *P*-value < 0.05 was used to determine the enriched GO and KEGG terms in these genes.

### Experimental Verification and Statistical Analysis

The 2^–ΔΔ*CT*^ formula was used to calculate qPCR relative quantification data. Internal control was β-actin or U6 small nuclear RNA. The student’s *t*-tests were employed to compare data by the SPSS (SPSS version 19.0, Inc., Chicago, IL, United States). All the primers for the qPCRs are presented in Supplementary Table S1.

### Cell Culture and Transfection

GC-1spg and TM3 cells were cultured in regular conditions (37°C, 5% CO2) with DMEM/high-glucose medium (Hyclone) in addition with 10% fetal-bovine serum (FBS, Gibco) and 1% penicillin/streptomycin (Hyclone). The cells were seeded into 6-well plate for treatment. Each well was seeded with 1 × 10^6^ cells (TM3) or 7 × 10^5^ cells (GC-1spg) and treated when the cell densities were 40%, including five treatment groups: MOCK, NC, mimics, iNC, and inhibitor (GenePharma, Shanghai, China). The transfection concentration was 50 nM, and were transfected by 3 μL Lipofectamine 2000 (Invitrogen, Carlsbad, CA, United States). The transfection method was referred to the protocol provide by Invitrogen. After 48 h of transfection, cells were collected for analysis with 0.25% trypsin (Gibco) trypsinized. Repeat at least three times for each experiment.

### CCK-8 Assays

Cells were seeded into 96-well plates, and each well was seeded with 8000 cells (TM3) or 5000 cells (GC-1spg) cells in 100 μL medium. The transfection concentrations of NC, mimics, iNC and inhibitor were 50 nM. After 48 h of treatment, 10 μL of Cell Counting Kit 8 (CCK-8, C0037, Beyotime Institute of Biotechnology) solution was added and incubation for 1 h at 37°C. Then, the absorbance at 450 nm was measured by a microplate reader.

### Annexin-V-FLUOS and PI Staining Assay

Cells were trypsinized and centrifuged at 450 *g* for 6 min, resuspended the cells with DPBS at room temperature, centrifuged again to remove the supernatant. Subsequently, cells were resuspended in a 200 μL binding buffer containing 2 μL PI and 2 μL annexin-V-FLUOS, incubated on ice for 30 min, and analyzed by flow cytometry (BD FACSAria III, BD Biosciences).

### TUNEL Staining Assay

*In Situ* Cell Death Detection Kit (Vazyme) was used to detect the apoptotic cells. The detected method was referred to the protocol provide by Vazyme. The nuclei were visualized using 4′,6′-Diamidino-2-phenylindole (DAPI, CWBIO). Digital images were captured with a fluorescence microscopy (Nikon Eclipse 80i, Tokyo, Japan).

### EdU Staining Assay

EdU kit (RiboBio, Guangzhou, China) was used to detect proliferation of the cells. The detected method was referred to the protocol. The nuclei were stained with Hoechst 3342 and digital images were captured by fluorescence microscopy (Nikon Eclipse 80i, Tokyo, Japan).

### Flow Cytometry Detect Cell-Cycle

Cells were seeded in 6-well plates and treated for 48 h, then trypsinized and centrifuged at 800 *g* for 5 min, resuspended the cells with chilled DPBS, and slowly added an appropriate amount of 100% absolute ethanol to achieve a final concentration of 70%, stored at −20°C. Subsequently, treated with the cell-cycle-staining kit (Multisciences, Hangzhou, China) and analyzed by flow cytometry (BD FACSAria III, BD Biosciences) (Jin et al., 2019).

### Western Blot

Western blots were carried out using antibody against Caspase 3 (Proteintech, 1:600), BAX (CST, 1:1000), Bcl2 (Proteintech, 1:500), PI3K (Santa, 1:200), AKT (Santa, 1:200), p-AKT (Santa, 1:200), RAP2B (Santa, 1:100), β-Actin (Proteintech, 1:6000), and PCNA (Proteintech, 1:200) according to the protocol described previously (Yang et al., 2019).

The cells were lysed with RIPA (P0013B, Beyotime Institute of Biotechnology) which containing 1 mmol/L PMSF (ST506, Beyotime Institute of Biotechnology). According to the size of the target protein, make separating gel and stacking gel with the corresponding concentration. The sample volume per well was 25 ng, and the electrophoresis voltage was 80 V and 110 V, respectively. Then the PVDF membrane was activated by methanol and semi-dry electrophoretic transfer was performed. The membrane followed by incubation with 5% skim-milk at room temperature for 2 h; the primary antibody was incubated at 4°C overnight; the secondary antibody (dilution ratio 1: 3000) was incubated at room temperature for 2 h after TBST cleaning. The membranes were stained with the reagents in Western Bright ECL Kit and visualized using Bio-Rad Chemidoc.

### Dual-Luciferase Reporter System Assay

Eight miR-205 candidate target genes were selected using TargetScan and miRWalk 2.0 databases combined with mRNA-seq data, which were BTBD3, CADM1, HS3ST1, NAA25, SLC35B3, SRSF10, and RAP2B. The 3′-UTR of target genes containing wild-type (WT) or mutant (Mut) in the seed region were cloned (the primers are presented in Supplementary Table S2). All amplified sequences were ligated to the psi-CHECK2 dual-luciferase reporter vector (Promega, Madison, AL, United States) using T4 DNA ligase (TaKaRa, Dalian, China) according to the manufacturer’s instructions. Sequencing was performed for validating all the constructs.

All the luciferase reporter plasmid vectors were transfected into HeLa cells by Lipofectamine 2000 along with miR-205 mimics or NC. The Renilla and Firefly luciferase activity was measured using the Dual-Glo Luciferase Assay System (Promega, Madison, AL, United States). All the experiments were performed in triplicates at least. The Renilla luciferase activity was employed for normalizing the signal value.

### Statistical Analysis

All experiments were repeat at least three times. All data were analyzed using SPSS 23.0 (SPSS Inc., Chicago, IL, United States) and were expressed as the means ± standard errors (SE). ANOVA with Tukey’s HSD *post hoc* test was applied to multigroup comparisons, whereas student’s *t* test was used for the two-group comparisons. When the *P* value was less than 0.05 (^∗^) or 0.01 (^∗∗^) the data were considered statistically significant.

## Results

### EDS Specifically Eliminate Pig Differentiated LCs

**FIGURE 1:**
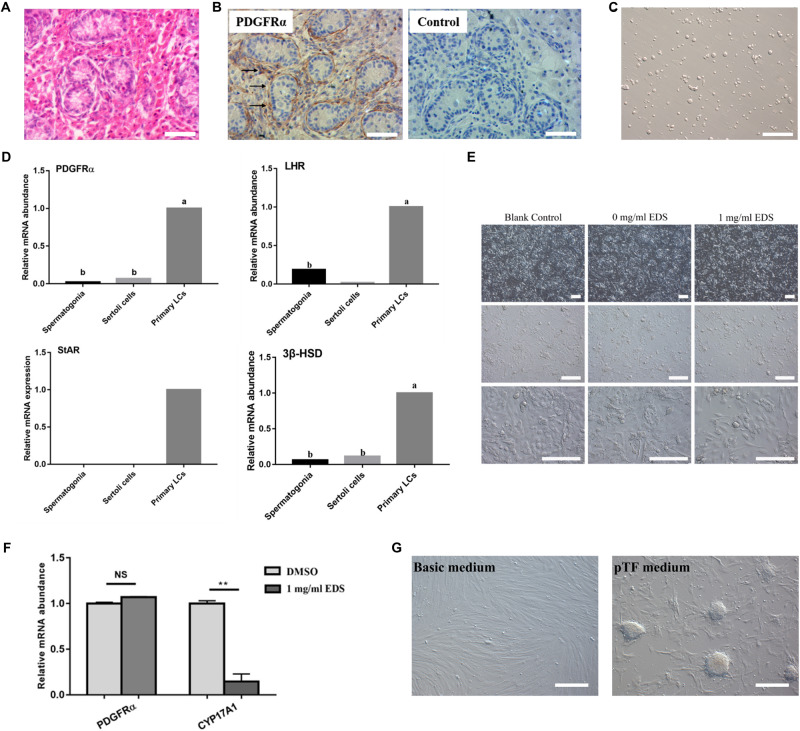
EDS can specifically eliminate pig differentiated LCs. **(A)** H&E staining of 7-day-old pig testes. **(B)** Immunohistochemical analysis of PDGFRα of 7-day-old pig testes, the black arrowheads indicated the PDGFRα-positive cells in testicular interstitium. **(C)** The morphology of LCs isolated from 7-day-old pig testis. **(D)** Expressions of SLCs and LC lineage marker genes in Spermatogonia cells, Sertoli cells and primary isolated LCs of pig. **(E)** Morphology of primary isolated LCs after EDS treatment. The primary LCs isolated from 7-day-old pig testes were treated with 1 mg/ml EDS for 24 h. **(F)** Expressions of PDGFRα and CYP17A1 of primary isolated pig LCs after EDS treatment as fold change relative to DMSO treated group. ** represent *P* < 0.01. NS means not significant. **(G)** Pig LCs was cultured for 12 days in basic medium and pTF (after EDS treatment) medium (bar = 50 μm). The two groups with different letters (a, b) mean *P* < 0.05, while the same letter means not significant.

The H&E staining showed that spindle-shaped cells were found in the testicular interstitium of 7-day-old pig testes (Figure 1A), and these cells could be stained with SLCs molecular marker, PDGFRα (Figure 1B). Subsequently, the primary pig LCs was enriched according to the protocol as our research group previously described (Figure 1C; Yu et al., 2017). Theoretically, the 7-day-old pig testes not only contain SLCs, but also include other Leydig lineage cells. Indeed, the primary isolated LCs did not merely express SLCs marker (PDGFRα), but also expressed LC lineage markers (LHR, StAR, and 3β-HSD) (Figure 1D). While the SCs marker SOX9 and SSCs marker PLZF were not detected (Yu et al., 2017), suggesting that there was no contamination with SCs and SSCs in pig LCs.

Ethane dimethane sulfonate was used to specifically eliminate differentiated rodent LCs (Ge et al., 2006). According to our previous study, EDS can eliminate pig ALCs. Meanwhile, we have screened various concentration and treated time, the optimal treatment concentration and time was 1.0 mg/mL and 24 h (Yu et al., 2017). In this study, 1.0 mg/mL EDS was used to treat primary pig LCs and the cell number was reduced after 24 h incubation (Figure 1E). In addition, the expression of PDGFRα has no significant differences with or without EDS treatment, while the expression of CYP17A1 was significantly decreased in the presence of EDS (Figure 1F). By calculating, our group’s study revealed that the percentage of pig differentiated LCs was approximately 23% in the primary isolated LCs, and the purity of primary isolated pig SLCs was over 77% (Yu et al., 2017). All these data indicated that 1.0 mg/mL EDS treatment for 24 h could specifically eliminate pig differentiated LCs.

Based on these findings, the primary isolated pig LCs were incubated with EDS for 24 h to eliminate pig differentiated LCs. Then these cells were cultured in the short-term pig SLCs culture system (Yu et al., 2017), and stem cell-like clones were formed 2 weeks later (Figure 1G). Short-term culture results further indicated that the isolated primary LCs contains SLCs, which was consistent with previous results (Yu et al., 2017).

Currently, few studies focus on the mechanism of mRNAs or miRNAs regulating differentiation of pig LCs. Thus, in this study, the newly isolated pig primary LCs named primary group (containing SLCs and differentiated LCs) and 1.0 mg/mL EDS treatment for 24 h LCs named EDS-treated group (almost exclusively SLCs) were collected for RNA-seq. We did not sequence SLC clones because collecting enough clones that can be sequenced was time consuming and difficult. The results also showed that EDS can eliminate pig differentiated LCs and the qRT-PCR results indicated that the expression of CYP17A1 decreased to a very low level. Therefore, the difference between the primary group (containing SLCs and differentiated LCs) and the EDS-treated group (almost exclusively SLCs) can reflect the expression spectrum of mRNAs and miRNAs related to pig LCs cell lineage differentiation, which can provide a basis for subsequent studies.

### Analysis of mRNA-seq Data and Validation of Differentially Expressed Genes

**FIGURE 2:**
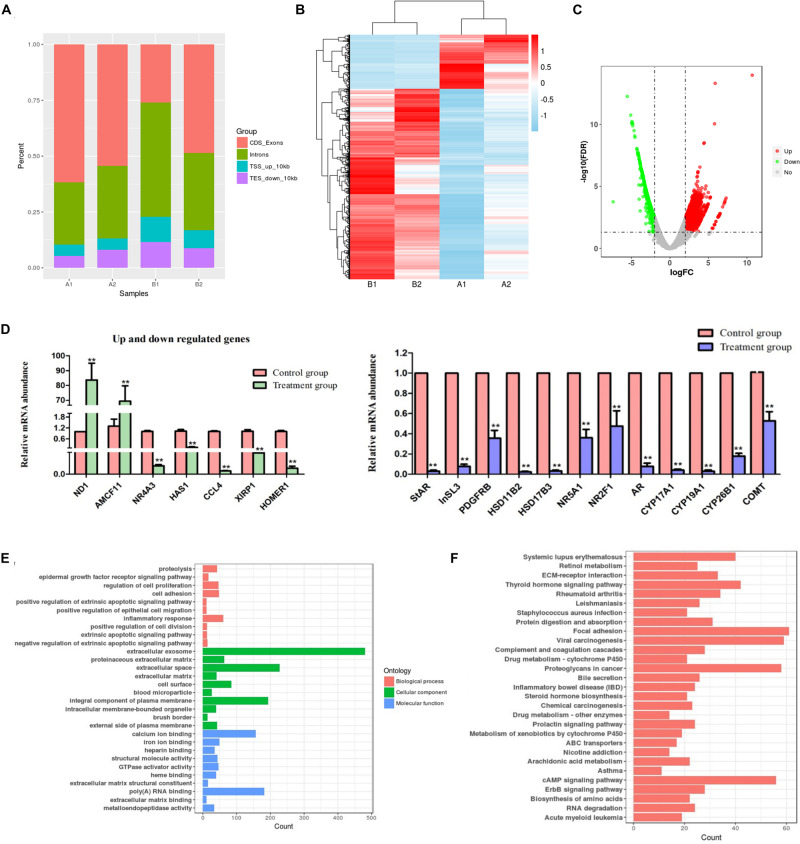
mRNA-seq data and differentially expressed genes analysis. **(A)** Percentage of reads mapped to different genome regions. CDS, coding sequence; TES, transcription end site; TSS, transcription start site. A1 and A2 represent primary group; B1 and B2 indicate EDS-treated group. **(B)** Clustering analysis of DEG. Up- and down- regulation are separately colored in red and blue. **(C)** Volcano plots showing the differently expressed genes in primary group compared to EDS-treated group. **(D)** The mRNA expression levels of up and down regulated genes and “steroid hormone biosynthesis” pathway related genes. The error bars are the S.E. of duplication. ** represent *P* < 0.01. **(E)** Significantly different top 10 GO terms correspond to different categories in primary group compared to EDS-treated group. **(F)** Significantly different top 30 KEGG terms correspond to different categories in primary group compared to EDS-treated group.

Four samples were used for the deep RNA sequencing method, four mRNA sequence files (two primary groups and two EDS-treated groups) were analyzed to obtain the global view of the transcriptome of pig LCs. On average, approximately 64% of reads were successfully mapped to the pig genome (Table 1). The proportion of reads that aligned to exonic regions was markedly lower in the EDS-treated group (26.05% and 48.66%) than in primary group (61.63% and 54.37%). Conversely, the percentage of reads that aligned to intron regions was dramatically higher at the EDS-treated group (Figure 2A). A pheatmap of DEGs and the volcano plots was produced based on the normalized reads in RNA-seq data (Figures 2B,C). Total 4,904 genes were significantly differently expressed (log2FC > 2 and *P* < 0.05). All DEGs were provided in Supplementary Table S3. Total 2,249 genes were up-regulated in the EDS-treated group compared to the primary group, whereas 2,645 genes were down-regulated. Top 10 DEGs that were up-regulated or down-regulated in the EDS-treated group compared to the primary group were shown in Tables 2, 3, respectively.

**TABLE 1:** Summary of reads mapping to the pig reference genome.

**Samples**	**A1**	**A2**	**B1**	**B2**
**Raw reads**	92,916,116	108,502,378	94,332,390	79,706,240
**Clean reads**	91,212,014	107,457,682	93,436,652	79,109,010
**Clean ratio**	98.17%	99.04%	99.05%	99.25%
**Mapped Reads**	65,152,606	69,332,338	51,363,550	51,382,018
**Mapped ratio**	71.43%	64.52%	54.97%	64.95%

*A1 and A2 represent primary group; B1 and B2 indicate EDS-treated group.*

**TABLE 2:** The top 10 up-regulated genes at the EDS-treated group compared to primary group.

**Gene symbol**	**A1**	**A2**	**B1**	**B2**	***P*-value**	**FDR**	**Significant (B vs. A)**
ADAMDEC1	2.893	6.143	809.117	113.533	7.34E-40	1.32E-35	up
MMP9	9.374	2.457	181.797	410.684	5.23E-33	4.71E-29	up
CYP1A1	0.231	3.440	25.680	147.593	2.13E-26	7.67E-23	up
AMCF-II	17.360	26.415	517.672	796.914	1.72E-25	5.17E-22	up
MMP1	24.304	39.193	496.884	754.558	3.29E-21	3.48E-18	up
CTSL	48.260	34.032	641.587	834.467	1.05E-19	7.54E-17	up
CCL17	0.116	0.369	22.011	34.060	5.06E-19	2.94E-16	up
NPM3	22.568	15.849	160.601	331.429	3.76E-18	1.74E-15	up
ND1	69.786	72.488	593.488	864.597	3.11E-14	5.90E-12	up
IL1RL1	4.745	4.546	76.632	35.370	8.82E-13	1.18E-10	up

*Differential expression standard: *P*-value ≤ 0.01, false discovery rate (FDR) ≤0.01; the RNA sequencing data were from 2 separated RNA samples (A1 and A2 represent primary group; B1 and B2 indicate EDS-treated group).*

**TABLE 3:** The top 10 down-regulated genes at the EDS-treated group compared to primary group.

**Gene symbol**	**A1**	**A2**	**B1**	**B2**	***P*-value**	**FDR**	**Significant (B vs. A)**
XIRP1	775.051	525.229	13.451	13.755	1.33E-29	7.97E-26	down
HOMER1	344.069	989.520	22.011	21.397	3.95E-25	1.02E-21	down
ATP1A2	329.950	259.973	13.859	10.917	6.61E-24	1.32E-20	down
HAS1	419.409	112.786	4.076	16.593	3.06E-24	6.88E-21	down
F3	955.479	1151.69611	21.6045	48.252	3.02E-23	4.94E-20	down
OGN	202.761	207.757	13.451	8.515	4.09E-21	4.09E-18	down
CCL21	176.606	42.633	5.299	4.803	8.42E-20	6.59E-17	down
CCL4	217.922	28.504	2.038	10.262	2.28E-19	1.52E-16	down
NR4A3	165.033	205.669	16.712	10.043	4.72E-18	2.12E-15	down
ETNK2	244.308	304.940	22.419	20.523	1.32E-17	5.42E-15	down

*Differential expression standard: *P*-value ≤ 0.01, false discovery rate (FDR) ≤0.01; the RNA sequencing data were from 2 separated RNA samples (A1 and A2 represent primary group; B1 and B2 indicate EDS-treated group).*

To validate the reliability of the mRNA-seq analysis, 19 candidate DEGs were selected to perform qRT-PCR, which included the significantly up/down-regulated genes, as well as the genes selected randomly according to the “steroid hormone biosynthesis” pathway (Figure 2D). The results showed that the expression of these DEGs between the primary group and EDS-treated group shared the same trend with the mRNA-seq data. Thus, these results suggested that the mRNA-seq data were believable in the study. Meanwhile, qRT-PCR results further confirmed almost no differentiated LCs existed in the EDS-treated group.

Gene ontology and KEGG pathway analysis of DEGs were detected. Based on the GO categories, a total of 279 clusters were annotated with GO terms (Figure 2E and Supplementary Table S4). GO annotation of these genes showed strong enrichment for terms related to extracellular space, extracellular exosome, integral component of plasma membrane, poly(A) RNA binding and calcium ion binding, suggesting the potential roles played by these DEGs in these functional areas. A total of 1,475 genes were annotated to 61 pathways in the KEGG database. What was worth mentioning is that the “steroid hormone biosynthesis” (ssc00140, 21 DEGs) pathway was also enriched at a high level. Top 30 significantly enriched KEGG pathways were shown in Figure 2F. All the KEGG pathways and related information were listed in Supplementary Table S5.

### Analysis of miRNA-seq Data and Detection of miR-205 Expression Profiles

**FIGURE 3:**
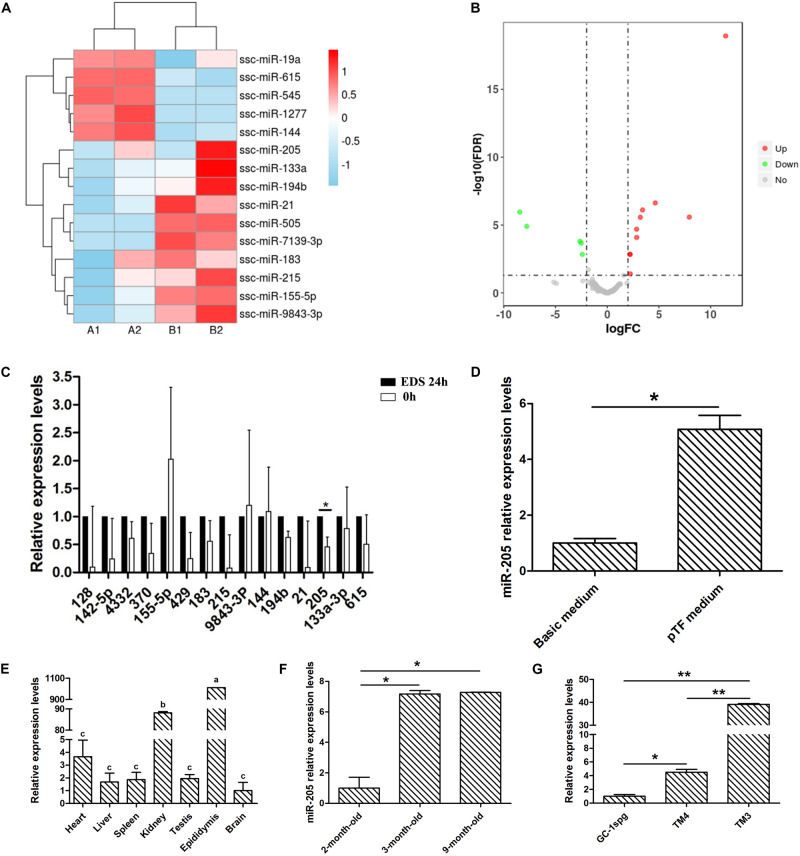
miRNA-seq data and expressio profiles analysis. **(A)** Clustering analysis of differently expressed miRNAs in primary group and EDS-treated group. **(B)** Volcano plots showing the differently expressed miRNAs in primary group compared to EDS-treated group. **(C)** Quantification of the miRNAs levels of differential expressed genes between primary group and EDS-treated group. **(D)** Quantification of the miR-205 level after cultured 2 weeks in basic medium and pTF medium, respectively. **(E)** Tissue expression profile of miR-205 in pig. **(F)** Timing expression profile of miR-205 in pig testis tissue. **(G)** Expression of miR-205 in TM3, TM4, and GC-1spg cell lines. * represent *P* < 0.05. ** represent *P* < 0.01. The two groups with different letters (a, b, c) mean *P* < 0.05, while the same letter means not significant.

By comparing the miRNA-seq data, 15 known miRNAs were found to be significantly differentially expressed between the primary group and the EDS-treated group (log2FC > 2 and *P* < 0.05). A clustered heat-map of differentially expressed miRNAs and the volcano plots were showed in Figures 3A,B, respectively. Total 10 miRNAs were significantly upregulated and 5 miRNAs were significantly down-regulated in the EDS-treated group compared with the primary group, in which miR-205 and miR-615 were the most up-regulated and down-regulated miRNAs in the EDS-treated group, respectively. To further validate the sequencing data, 15 miRNAs were selected from differentially expressed miRNAs for qRT-PCR validation. The results of the validation showed that the expression level of miR-205 in the EDS-treated group was significantly higher than that in the primary group (*P* < 0.05) (Figure 3C), which was consistent with the sequencing data.

Combined with previous experimental results, EDS treatment of pig primary LCs could eliminate differentiated LCs and retained SLCs. Therefore, the expression spectrum of mRNA and miRNA before and after EDS treatment can partly reflect the differences during LCs differentiation. Subsequently the miR-205 expression level was detected in SLCs clones for 2 weeks cultured in pTF and differentiated LCs for 2 weeks cultured in basal medium (Figure 3D; Yu et al., 2017). Quantitative results showed that the expression of miR-205 in SLCs clones was significantly higher than that in differentiated LCs, which was consistent with the miRNA-seq data. Therefore, miR-205 was selected for further study.

The expression profiles of miR-205 were explored. It was expressed in all detected tissues and significantly highly expressed in epididymis (Figure 3E). With the development of testis, miR-205 expression was significantly increased (Figure 3F). The miR-205 expression was also validated in three different cell lines, mouse type B spermatogonia cell line (GC-1spg), mouse Sertoli cell line (TM4), and mouse Leydig progenitor cell line (TM3). The results showed that miR-205 was highly expressed in TM3 compare with the other two cell lines (Figure 3G).

### MiR-205 Did Not Affect Cell Steroidogenesis and Proliferation

**FIGURE 4:**
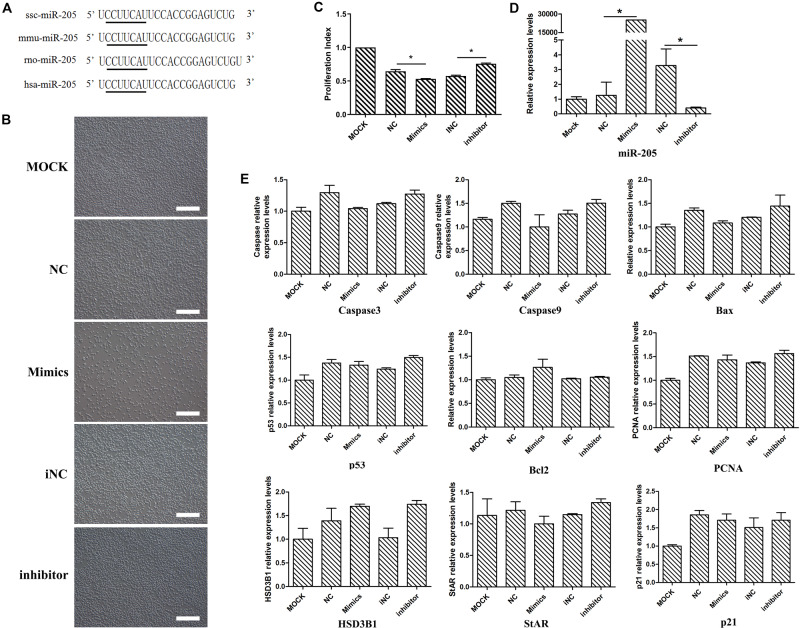
The phenotype and several genes expression levels after miR-205 treatment in TM3 cells. **(A)** The sequence of pig, mouse, rat, and human miR-205 sequences. Black underline represents the seed sequence. **(B)** The phenotype produced by overexpress or knock down miR-205 in TM3 cells (bar = 50 μm). **(C)** Cell viability of TM3 cells after miR-205 treatment for 48 h. **(D)** miR-205 expression level after overexpression or knockdown for 48 h. **(E)** Apoptosis, proliferation and Leydig cell differentiation related genes expression after miR-205 treatment for 48 h. * represent *P* < 0.05.

The miR-205 sequences of mouse, rat, human and pig were obtained from the miRBase database. According to sequence alignment, the mature miR-205 sequences of pig (ssc-miR-205), mouse (mmu-miR-205), and human (hsa-miR-205) were identical (Figure 4A), suggesting miR-205 was highly conserved among different species. Given the low transfection efficiency in primary stem cells and the unavailability of porcine Leydig cell line, subsequent investigations were performed on mouse Leydig progenitor cell line TM3.

In order to explore the function of miR-205, miR-205 mimics and inhibitor were transfected. After miR-205 overexpressed for 48 h, the number of TM3 was significantly reduced, while inhibiting miR-205 expression, the number of cells did not change significantly (Figure 4B). Subsequently, the effect of miR-205 on the viability of TM3 cells was performed by CCK-8 analysis. After overexpression miR-205 the cell viability was significantly down-regulated compared with NC group, while after knock-down miR-205 expression the cell viability was up-regulated compared with iNC group (Figure 4C). By quantitative analysis, the miR-205 expression level was significantly up-regulated in mimics group compared with NC group, and inhibitor could significantly down-regulated miR-205 expression in TM3 cells (Figure 4D), suggesting the subsequent experiments can be performed.

In rodents, the ALCs lineage can be divided into four distinct stages: stem cell, progenitor cell, immature cell and adult cell (Ye et al., 2017). TM3 cell line has been established from testis of 11 to 13 days mice, and LCs at this time are in an undifferentiated state which belonged to progenitor Leydig cell. In these stages, the key genes involve in T synthesis, including HSD3B1 and StAR, are not expressed. Therefore, the expression of these steroidogenesis related genes serve as one of the indicators of Leydig cell differentiation. To determine whether miR-205 affects TM3 cell steroidogenesis, the expressions of HSD3B1 and StAR genes were detected. The expressions of HSD3B1 and StAR genes did not change with up/down-regulation of miR-205 (Figure 4E), suggesting that miR-205 may not affect the steroidogenesis of LCs. According to the qRT-PCR, CCK-8 results and cell phenotype, miR-205 may be involved in the regulation of cell proliferation or apoptosis.

**FIGURE 5:**
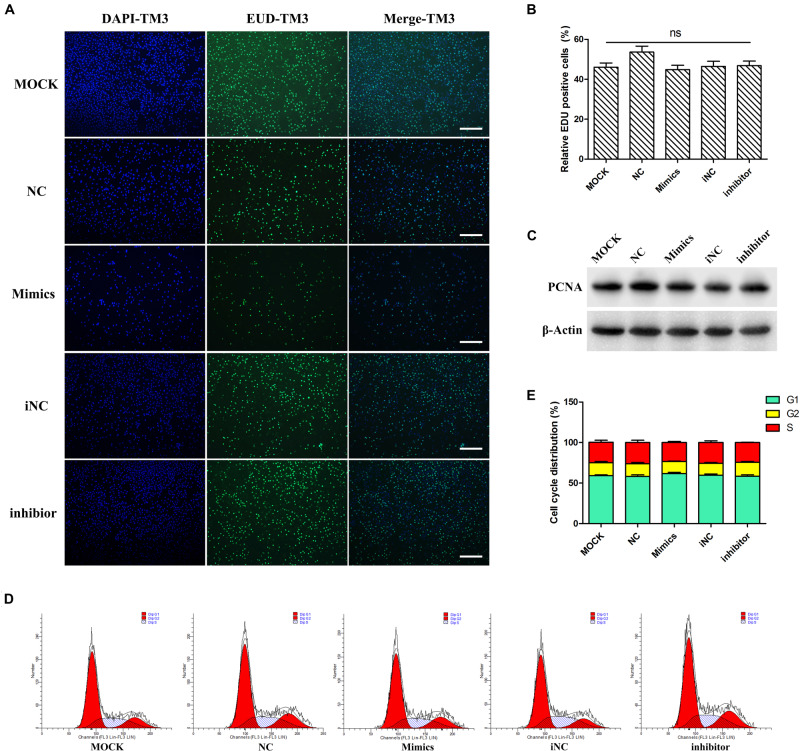
miR-205 had no significant effect on TM3 cells proliferation. **(A,B)** EdU assay was carried out after transfection for 48 h. And the EDU positive cells were calculated (bar = 50 μm). **(C)** PCNA protein expression level after overexpression or knockdown miR-205 in TM3 cells. **(D,E)** Cell cycle distribution was performed by flow cytometer after transfection for 48 h.

At mRNA level, miR-205 did not affect PCNA and p21 gene expression (Figure 4E). The EDU experiments were performed on TM3 cells, there have no difference comparing the percentage of positive EDU cells (Figures 5A,B). Results of WB analysis revealed that overexpression or knock-down miR-205 did not affect the expression of PCNA protein in TM3 cells (Figure 5C). To further investigate the effect of miR-205 on proliferation, flow cytometry detected the distribution of cell cycle, and the results showed no significant difference among different groups (Figures 5D,E).

To detect whether the regulatory function of miR-205 was broad-spectrum, the experiments were also performed on GC-1spg cell. The phenotype and cell viability of GC-1spg cells was similar to TM3 cells (Supplementary Figure S1). qRT-PCR results showed that up/down-regulation of miR-205 did not affect the CCND1 and p21 gene expression (Supplementary Figure S1). In addition, EDU, flow cytometry and PCNA protein expression experiments showed that miR-205 had no influence on GC-1spg cell proliferation (Supplementary Figure S2). Taken together, all these results suggested that miR-205 did not affect TM3 and GC-1spg cell proliferation.

### MiR-205 Induced Cell Apoptosis

**FIGURE 6:**
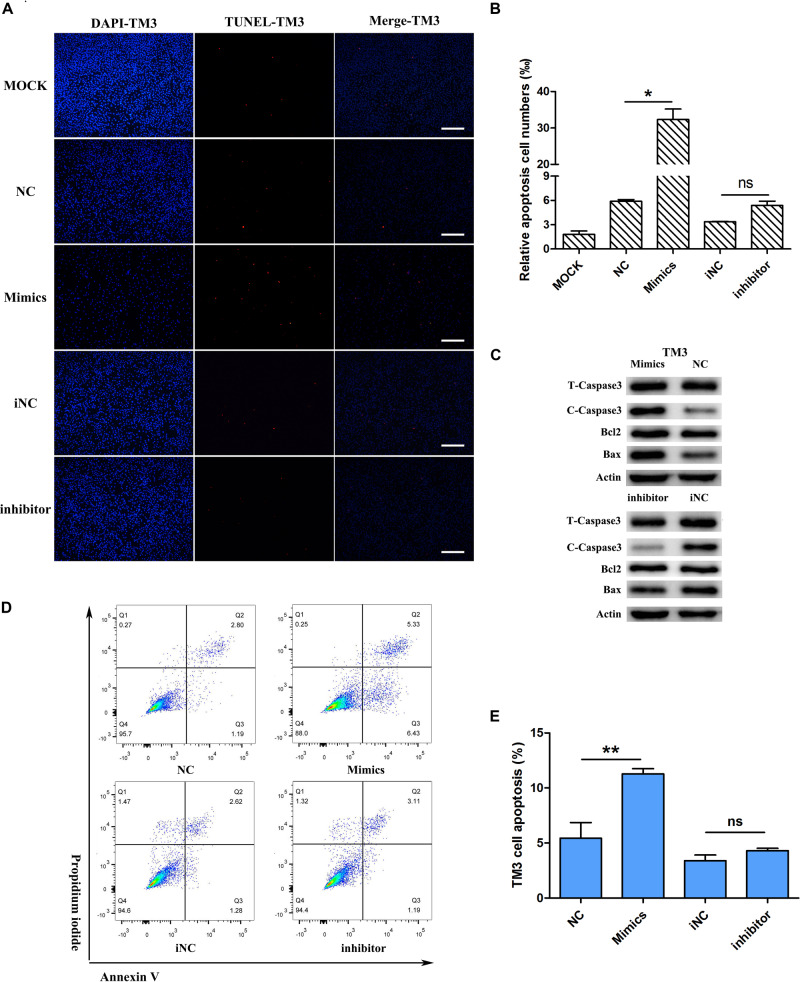
miR-205 induces TM3 cell apoptosis. **(A,B)** TUNEL assay was carried out after transfection for 48 h. And the TUNEL positive cells were calculated (bar = 50 μm). **(C)** apoptosis-related protein expression after transfection miR-205 in TM3 cells. **(D)** Annexin V-FITC/PI analysis after overexpression or knockdown miR-205 in TM3 cells. Cells apoptosis phase distribution were analyzed by flow cytometry. **(E)** Cells apoptosis index were analyzed. * represent *P* < 0.05. ** represent *P* < 0.01. ns means not significant.

Considering that miR-205 had no significant effect on the proliferation of TM3 and GC-1spg cells, this study further examined whether miR-205 triggers apoptosis. First, the expression of apoptosis-related genes was detected. The results showed that up/down-regulation of miR-205 did not affect the expression of apoptotic genes, including Caspase3, Caspase9, BAX, and Bcl2 in TM3 cells (Figure 4E). TUNEL staining was performed on TM3 cell, the number of positive cells were counted. Overexpression of miR-205 significantly increased the number of TUNEL positive cells, while reduction of miR-205 had no influence on TUNEL positive cells compare with iNC group (Figures 6A,B).

The expression of apoptosis-related genes was detected at the protein level. In TM3 cells, overexpression of miR-205 increased the expression of BAX and cleaved-caspase3, and there was no significant difference of Bcl2 expression. Reduction of miR-205 expression reduced BAX and cleaved-caspase3 protein expression (Figure 6C). To further confirm the apoptotic phenotype, apoptotic flow cytometry assay was performed. The results showed that overexpression of miR-205 caused a significant increase in apoptotic cells, but inhibition of miR-205 expression did not attenuate apoptosis (Figures 6D,E).

These experiments were also performed in GC1-spg cells. The expression of apoptotic genes and autophagy-related genes, including Becn1, LC3, and Atg12, did not change (Supplementary Figure S1). In GC-1spg cells, up-regulation of miR-205 reduced Bcl2 protein expression and increased expression of cleaved-caspase3. Down-regulation of miR-205 increased Bcl2 and reduced cleaved-caspase3 expression, whereas the expression of cleaved-caspase9 had not significantly different between the treated and control groups (Supplementary Figure S3). The TUNEL and flow cytometry assay of GC-1spg cells was similar to TM3 cells (Supplementary Figure S3). These results indicated that the phenotype of miR-205-induced cell number reduction was the result of apoptosis induction.

### RAP2B Was a Target Gene of miR-205

**FIGURE 7:**
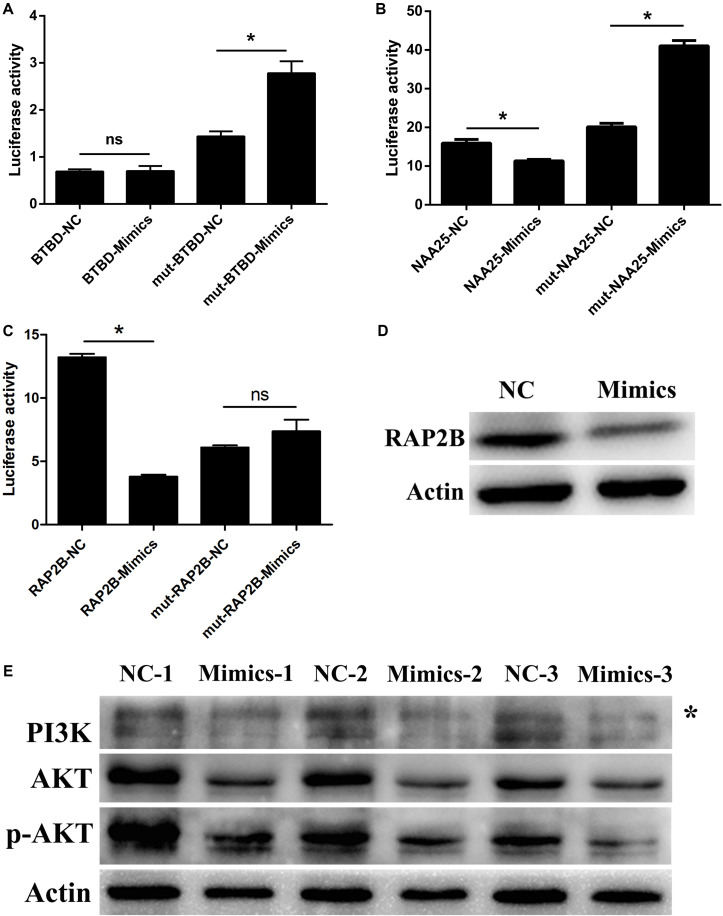
miR-205 induces Leydig cell apoptosis by targeting RAP2B via the PI3K/AKT signaling pathway. **(A–C)** The results of Dual luciferase reporter assay of three potential targets of miR-205 predicted by bioinformatics analysis. **(D)** RAP2B protein expression after transfection miR-205 in TM3 cell. **(E)** PI3K, AKT and p-AKT protein expression after transfection miR-205 in TM3 cell. Asterisk indicates non-specific band.

Through TargetScan and miRWalk 2.0 databases combined with mRNA-seq data, seven candidate target genes were selected, including BTBD3, CADM1, HS3ST1, NAA25, SLC35B3, SRSF10, and RAP2B. Due to the difficulty in the amplification of the mutant (Mut) sequence, only three genes’ Mut vectors (BTBD3, NAA25, and RAP2B) were constructed (Supplementary Figure S4). The fluorescence of RAP2B-WT vector after treatment with miR-205 was significantly lower than that of NC group. Meanwhile, there was no significant change in the RAP2B-Mut vector after treated with miR-205 or NC (Figures 7A–C). The fluorescence activity of the BTBD3-Mut and NAA25-Mut vectors were significantly increased after miR-205 treatment, which did not meet the expectations of dual fluorescence reporter assays. Meanwhile, the WB results showed that miR-205 specifically suppress RAP2B protein expression in TM3 cell (Figure 7D). The results indicated that RAP2B was a target gene for miR-205.

### MiR-205 Inhibited PI3K/AKT Pathway

Rap2B is a member of the Ras family belonging small GTP-binding proteins. Previous study revealed that Ras can active PI3K/AKT pathway (Yoshikawa et al., 2017). According to these results, the PI3K, AKT, and p-AKT expression were explored. Because the aim of this study was to detect the functional regulation mechanism of LCs, the protein expression was only detected in TM3 cell. The results showed that miR-205 overexpression decrease the expression of PI3K, AKT, and p-AKT (Figure 7E).

## Discussion

Testosterone is critical for somatic cell function, development of the male reproductive system, male phenotype and sexual behavior maintenance (Smith and Walker, 2014). T is secreted by ALCs which are derived from SLCs. Pigs are important mammalian model and economic animal, however, few studies have focused on pig LCs. In 2017, our research group firstly established the *in vitro* short-term culture system for pig SLCs (Yu et al., 2017).

The 7-day-old pig testes had PDGFRα positive SLCs in the testicular interstitium. Theoretically, the 7-day-old pig testes not only contained SLCs, but also included other LC lineage cells. Indeed, the primary isolated LCs could not only expresse makers of SLCs (PDGFRα), but also expressed LC lineage markers (LHR, StAR, and 3β-HSD). In rodents, there is another LC population: fetal Leydig cells (FLCs) (Chen H. et al., 2017). Around gestational days 11–12, FLCs are developed between the testis cords, and remain in the testicular interstitium after birth. Subsequently, FLCs are gradually involuted, retaining a few in the adult testis (Huhtaniemi and Pelliniemi, 1992; Wen et al., 2016; Zirkin and Papadopoulos, 2018). Recently, Shima et al. (2015) demonstrated that the FLCs, which contributed to androgen production and survive through the adulthood, could be minimal because of their relatively low number compared to the adult stage (Zirkin and Papadopoulos, 2018). There are some questions need to be explained in future experiments. For example, whether FLCs exist in the primary isolated pig LCs, and if they exist, how to identify the proportion of these extremely rare FLCs in addition to the function of FLCs in the development of pig.

In Jackson et al. (1959) first described the antifertility effects of EDS, which is an alkylating sulfonic ester (Zhang M. et al., 2017). Molenaar et al. (1985) demonstrated that EDS, with a dose of 75 mg/kg body weight through injection, could destroy not only mature LCs, but also those LC precursors that were able to differentiate rapidly into recognizable LCs within 4-day treatment in rats (Teerds et al., 1988). This conclusion was further confirmed by other researchers (Zang et al., 2017; Zhang M. et al., 2017). In addition, EDS had effects on ALCs in the testis of the guinea pig (Kerr et al., 1987), frog (Minucci et al., 1990), rabbit (Laskey et al., 1994), and lizard (Minucci et al., 1995). In this study, EDS could eliminate differentiated LCs in pigs, which was accompanied with a decreased expression of CYP17A1, whereas the expression of PDGFRα did not change. The molecular pathway mechanisms underlying EDS action on LCs were related to intracellular glutathione (Kelce and Zirkin, 1993), activation of the Fas receptor (Taylor et al., 1999), oxidative stress (Lee et al., 2012), involvement of Bcl-2 family members (Taylor et al., 1998), or alkylation of proteins (Rommerts et al., 1985). In this study, we first uncovered that miR-205 was also involved in the process of EDS to eliminate differentiated LCs. These results will deepen the understanding of the mechanism of EDS-induced pig LC elimination.

In this study, the primary pig LCs (containing SLCs and differentiated LCs) and EDS-treated LCs (almost exclusively SLCs) were sequenced. The differentially expressed mRNAs and miRNAs between the primary and the EDS-treated group may play roles in the regulation of pig LCs differentiation. The mRNA-seq data uncovered 4,904 DEGs between the two types of samples. The genes related to “steroid hormone biosynthesis” pathway were reduced after EDS treatment. In 2014, the mRNA transcripts levels of adult male rats testes with butane dimethane sulfonate (eliminate germ cell population) and EDS (ablate LC population) co-treated were measured using microarrays (O’Shaughnessy et al., 2014). The expression of StAR and CYP17A1 showed a gradual decline after EDS treatment, which was consistent with the results in this study. This analysis results further validated that EDS could eliminate the differentiated pig LCs.

Among the 4,904 DEGs, the genes related to steroid hormone biosynthesis were significantly decreased. CYP19A1 encodes a member of the cytochrome P450 superfamily of enzymes. The cytochrome P450 proteins are monooxygenases which catalyze many reactions involved in drug metabolism and synthesis of cholesterol, steroids and other lipids (DuPree et al., 2004). Ge et al. (2005) also found that the expression levels of CYP17A1 and HSD11B2 were increased by at least twofold with the proliferation and differentiation of LCs from PLCs to ILCs, and then to ALCs. In addition, Purves-Tyson et al. (2012) reported that T could increase the mRNA expression level of COMT (catechol-O-methyl transferase), which was consistent with the results in this study where the expression level of COMT was decreased in the EDS-treated group compared with that of the primary group.

By analyzing the miRNA-seq data, miR-205 was thought to play a role in pig LCs differentiation regulation. However, experiments on cell lines had validated that miR-205 induced apoptosis without affecting cell differentiation or proliferation. According to previous reports, miR-205 regulated various biological processes, including proliferation, migration, apoptosis, and epithelial to mesenchymal transition. It has been reported that miR-205 can promote cell proliferation by inhibiting SMAD4 gene expression in non-small cell lung cancer cells and promote tumor growth in nude mice (Zeng et al., 2017). In contrast, Chen et al. (2018) showed that miR-205 inhibited the growth and migration of neuroblastoma by its target gene CREB1 which further regulated the expression of downstream proliferation genes. In this study, miR-205 did not affect TM3 and GC-1spg cell proliferation. Previous studies had shown that miR-205 was implicated in apoptosis. Zhang et al. (2018) found that miR-205 expression was significantly higher in atretic follicle than healthy follicles. Exogenous overexpression of miR-205 in mouse granulosa cells promoted apoptosis and increased caspase-3/9 activity. The expression of miR-205 was increased in rat cavernous smooth muscle cells, which inhibited cell proliferation and promoted apoptosis by targeting AR (Wen et al., 2019). Taking those studies and ours together, miR-205 plays a role in promoting apoptosis.

Through luciferase reporter and WB assays, RAP2B was identified as a target gene for miR-205. Rap2B highly expressed in various human tumors, participating tumor cell proliferation, migration and invasion regulation (Di et al., 2016; Zhang L. et al., 2017). Previous study detected that Rap2B promoted renal cell carcinoma angiogenesis via PI3K/AKT signaling pathway. In this study, miR-205 overexpression inhibited PI3K, Akt and p-AKT expression. Thus, we deduced that miR-205 degraded PI3K/AKT signaling pathway via inhibiting RAP2B expression. It was reported that activation of PI3K/AKT pathway broadly benefited cell survival (Su et al., 2017; Yoshikawa et al., 2017). Thus, the apoptosis phenotype induced by miR-205 overexpression may be caused by PI3K/AKT signaling pathway disturbance.

## Conclusion

(1) The most up-regulated miRNA in the EDS treatment group was miR-205 which was highly expressed in pig SLCs clones compared with differentiated LCs.
(2) Overexpression of miR-205 induced TM3 and GC-1spg cells apoptosis.
(3) RAP2B was a target gene of miR-205.
(4) miR-205 inhibited PI3K/Akt pathway.

## Data Availability Statement

All of the raw sequence data were submitted to the NCBI SRA database: SRR11625159, SRR11625160, SRR11625161, SRR11625162, SRR11625155, SRR11625156, SRR11625157, and SRR11625158.

## Ethics Statement

The animal study was reviewed and approved by the Faculty Animal Policy and Welfare Committee of Northwest A&F University (NWAFAC1008).

## Author Contributions

YC, CP, and XL designed the experiment. RC, SY, and YZ completed mRNA sequencing and experimental verification. YC and MC completed miRNA sequencing and experimental verification. LM and WY performed the sequencing data analysis. YC, WZ, and WD wrote the manuscript.

## Conflict of Interest

The authors declare that the research was conducted in the absence of any commercial or financial relationships that could be construed as a potential conflict of interest.

## Footnotes

1 http://www.geneontology.org

2 http://david.abcc.ncifcrf.gov

## Supplementary Material

FIGURE S1 The phenotype and several genes expression levels after miR-205 treatment in GC-1spg cells. **(A)** The phenotype produced by overexpress or knock down miR-205 in GC-1spg cells (bar = 50 μm). **(B)** Cell viability of GC-1spg cells after miR-205 treatment. **(C)** miR-205 expression level after overexpress or knock down treatment for 48 h. **(D)** Apoptosis, proliferation and autophagy related genes expression after miR-205 treatment for 48 h. ^∗^ represent *P* < 0.05. ns means not significant.

FIGURE S2 miR-205 had no significant effect on GC-1spg cells proliferation. **(A,B)** EdU assay was carried out after transfection for 48 h (bar = 50 μm). **(C)** PCNA protein expression level after overexpression or knockdown miR-205. Cell cycle distribution were performed by flow cytometer after transfection for 48 h.

FIGURE S3 miR-205 induces GC-1spg cells apoptosis. **(A,B)** TUNEL assay was carried out after transfection for 48 h (bar = 50 μm). **(C)** apoptosis-related protein expression after transfection. **(D)** Annexin V-FITC/PI analysis after overexpression or knockdown miR-205 in GC-1spg cells. Cells apoptosis phase distribution were analyzed by flow cytometry. **(E)** Cells apoptosis index were analyzed. ^∗^ represent *P* < 0.05. ^∗∗^ represent *P* < 0.01. ns means not significant.

FIGURE S4 The sequencing results of BTBD3, NAA25, and RAP2B dual-luciferase reporter vector constructs. Red box indicates the mutated sequence.

TABLE S1 Primer sequences for genes designed and used in this study.

TABLE S2 Primers for amplify 3′-UTR of miR-205 target genes.

TABLE S3 Differential expressed genes between the EDS-treated group and primary group.

TABLE S4 GO terms of DEGs between the EDS-treated group and primary group.

TABLE S5 KEGG pathway of DEGs between the EDS-treated group and primary group.

## Abbreviations

ALCs: adult Leydig cells
CYP17A1: cytochrome P450 family 17 subfamily A member 1
DEGs: differentially expressed genes
DMSO: dimethyl sulfoxide
EDS: ethane dimethane sulfonate
FBS: fetal bovine serum
GO: Gene Ontology
HSD3B1: 3 beta- and steroid delta-isomerase 1
ILCs: immature Leydig cell
KEGG: Kyoto Encyclopedia of Gene and Genome pathway
LCs: Leydig cells
PDGFR α: platelet-derived growth factor receptor α
PLCs: progenitor Leydig cells
pTF: testicular fluid of piglets
qPCR: quantitative real-time PCR
SCs: Sertoli cells
SLCs: stem Leydig cells
SSCs: spermatogonial stem cells
StAR: Steroidogenic Acute Regulatory Protein
T: testosterone.

## References

[B1] Albert O. Huang J. Y. Aleksa K. Hales B. F. Goodyer C. G. Robaire B. (2018). Exposure to polybrominated diphenyl ethers and phthalates in healthy men living in the greater montreal area: a study of hormonal balance and semen quality. *Environ. Int.* 116 165–175. 10.1016/j.envint.2018.04.012 29684825

[B2] Bergfelder-Druing S. Grosse-Brinkhaus C. Lind B. Erbe M. Schellander K. Simianer H. (2015). A genome-wide association study in large white and landrace pig populations for number piglets born alive. *PLoS One* 10:e0117468. 10.1371/journal.pone.0117468 25781935 PMC4363374

[B3] Bolger A. M. Lohse M. Usadel B. (2014). Trimmomatic: a flexible trimmer for illumina sequence data. *Bioinformatics* 30 2114–2120. 10.1093/bioinformatics/btu170 24695404 PMC4103590

[B4] Chen H. Ge R. S. Zirkin B. R. (2009). Leydig cells: from stem cells to aging. *Mol. Cell. Endocrinol.* 306 9–16. 10.1016/j.mce.2009.01.023 19481681 PMC2749461

[B5] Chen H. Wang Y. Ge R. Zirkin B. R. (2017). Leydig cell stem cells: Identification, proliferation and differentiation. *Mol. Cell. Endocrinol.* 445 65–73. 10.1016/j.mce.2016.10.010 27743991 PMC5346484

[B6] Chen R. Du J. Ma L. Wang L. Q. Xie S. S. Yang C. M. (2017). Comparative micrornaome analysis of the testis and ovary of the chinese giant salamander. *Reproduction* 154 169–179. 28630098 10.1530/REP-17-0109

[B7] Chen S. Jin L. Nie S. Han L. Lu N. Zhou Y. (2018). Mir-205 inhibits neuroblastoma growth by targeting camp-responsive element-binding protein 1. *Oncol. Res.* 26 445–455. 10.3727/096504017x14974834436195 28653600 PMC7844742

[B8] Di J. Cao H. Tang J. Lu Z. Gao K. Zhu Z. (2016). Rap2b promotes cell proliferation, migration and invasion in prostate cancer. *Med. Oncol.* 33:58. 10.1007/s12032-016-0771-7 27154636

[B9] DuPree M. G. Mustanski B. S. Bocklandt S. Nievergelt C. Hamer D. H. (2004). A candidate gene study of cyp19 (aromatase) and male sexual orientation. *Behav. Genet.* 34 243–250. 10.1023/b:bege.0000017870.77610.52 14990865

[B10] Friedlander M. R. Mackowiak S. D. Li N. Chen W. Rajewsky N. (2012). Mirdeep2 accurately identifies known and hundreds of novel microrna genes in seven animal clades. *Nucleic Acids Res.* 40 37–52. 10.1093/nar/gkr688 21911355 PMC3245920

[B11] Gao F. Li G. Liu C. Gao H. Wang H. Liu W. (2018). Autophagy regulates testosterone synthesis by facilitating cholesterol uptake in leydig cells. *J. Cell Biol.* 217 2103–2119. 10.1083/jcb.201710078 29618492 PMC5987723

[B12] Ge R. S. Dong Q. Sottas C. M. Chen H. Zirkin B. R. Hardy M. P. (2005). Gene expression in rat leydig cells during development from the progenitor to adult stage: a cluster analysis. *Biol. Reprod.* 72 1405–1415. 10.1095/biolreprod.104.037499 15716394

[B13] Ge R. S. Dong Q. Sottas C. M. Papadopoulos V. Zirkin B. R. Hardy M. P. (2006). In search of rat stem leydig cells: identification, isolation, and lineage-specific development. *Proc. Natl. Acad. Sci. U.S.A.* 103 2719–2724. 10.1073/pnas.0507692103 16467141 PMC1413776

[B14] Ge R. S. Hardy M. P. (1998). Variation in the end products of androgen biosynthesis and metabolism during postnatal differentiation of rat leydig cells. *Endocrinology* 139 3787–3795. 10.1210/endo.139.9.6183 9724031

[B15] Heidari B. Rahmati-Ahmadabadi M. Akhondi M. M. Zarnani A. H. Jeddi-Tehrani M. Shirazi A. (2012). Isolation, identification, and culture of goat spermatogonial stem cells using c-kit and pgp9.5 markers. *J. Assist. Reprod. Genet.* 29 1029–1038. 10.1007/s10815-012-9828-5 22782689 PMC3492579

[B16] Huang X. Zhang B. Wu L. Zhou Y. Li Y. Mao X. (2019). Association of exposure to ambient fine particulate matter constituents with semen quality among men attending a fertility center in china. *Environ. Sci. Technol.* 53 5957–5965. 10.1021/acs.est.8b06942 31013428

[B17] Huhtaniemi I. Pelliniemi L. J. (1992). Fetal leydig cells: cellular origin, morphology, life span, and special functional features. *Proc. Soc. Exp. Biol. Med. Soc. Exp. Biol. Med.* 201 125–140. 10.3181/00379727-201-43493 1409728

[B18] Inoue M. Baba T. Morohashi K. I. (2018). Recent progress in understanding the mechanisms of leydig cell differentiation. *Mol. Cell. Endocrinol.* 468 39–46. 10.1016/j.mce.2017.12.013 29309805

[B19] Jackson H. Fox B. W. Craig A. W. (1959). The effect of alkylating agents on male rat fertility. *Br. J. Pharmacol. Chemother.* 14 149–157. 10.1111/j.1476-5381.1959.tb01375.x 13662565 PMC1481789

[B20] Jin Y. Wang J. Zhang M. Zhang S. Lei C. Chen H. (2019). Role of bta-mir-204 in the regulation of adipocyte proliferation, differentiation, and apoptosis. *J. Cell. Physiol.* 234 11037–11046. 10.1002/jcp.27928 30697738

[B21] Kelce W. R. Zirkin B. R. (1993). Mechanism by which ethane dimethanesulfonate kills adult rat leydig cells: involvement of intracellular glutathione. *Toxicol. Appl. Pharmacol.* 120 80–88. 10.1006/taap.1993.1089 8390114

[B22] Kerr J. B. Knell C. M. Abbott M. Donachie K. (1987). Ultrastructural analysis of the effect of ethane dimethanesulphonate on the testis of the rat, guinea pig, hamster and mouse. *Cell Tissue Res.* 249 451–457. 3621309 10.1007/BF00215530

[B23] Klinefelter G. R. Hall P. F. Ewing L. L. (1987). Effect of luteinizing hormone deprivation in situ on steroidogenesis of rat leydig cells purified by a multistep procedure. *Biol. Reprod.* 36 769–783. 10.1095/biolreprod36.3.769 3496123

[B24] Laskey J. W. Klinefelter G. R. Kelce W. R. Ewing L. L. (1994). Effects of ethane dimethanesulfonate (eds) on adult and immature rabbit leydig cells: comparison with eds-treated rat leydig cells. *Biol. Reprod.* 50 1151–1160. 10.1095/biolreprod50.5.1151 8025172

[B25] Le Moal J. Rolland M. Goria S. Wagner V. De Crouy-Chanel P. Rigou A. (2014). Semen quality trends in French regions are consistent with a global change in environmental exposure. *Reproduction* 147 567–574. 10.1530/rep-13-0499 24567426

[B26] Lee E. H. Oh J. H. Lee Y. S. Park H. J. Choi M. S. Park S. M. (2012). Gene expression analysis of toxicological pathways in tm3 leydig cell lines treated with ethane dimethanesulfonate. *J. Biochem. Mol. Toxicol.* 26 213–223. 10.1002/jbt.21409 22711419

[B27] Lo K. C. Lei Z. Rao, Ch V. Beck J. Lamb D. J. (2004). De novo testosterone production in luteinizing hormone receptor knockout mice after transplantation of leydig stem cells. *Endocrinology* 145 4011–4015. 10.1210/en.2003-1729 15123536

[B28] Minucci S. Fasano S. Di Matteo L. Chieffi Baccari G. Pierantoni R. (1990). Morphological and hormonal changes in the frog, rana esculenta, testis after administration of ethane dimethane sulfonate. *Gen. Comp. Endocrinol.* 79 335–345. 10.1016/0016-6480(90)90063-r 2177018

[B29] Minucci S. Fasano S. Marmorino C. Chieffi P. Pierantoni R. (1995). Ethane 1,2-dimethane sulfonate effects on the testis of the lizard, podarcis s. sicula raf: morphological and hormonal changes. *Gen. Comp. Endocrinol.* 97 273–282. 10.1006/gcen.1995.1027 7789742

[B30] Molenaar R. de Rooij D. G. Rommerts F. F. Reuvers P. J. van der Molen H. J. (1985). Specific destruction of leydig cells in mature rats after *in vivo* administration of ethane dimethyl sulfonate. *Biol. Reprod.* 33 1213–1222. 10.1095/biolreprod33.5.1213 3000465

[B31] Morris A. J. Taylor M. F. Morris I. D. (1997). Leydig cell apoptosis in response to ethane dimethanesulphonate after both *in vivo* and *in vitro* treatment. *J. Androl.* 18 274–280. 9203055

[B32] Ohno S. Nakajima Y. Nakajin S. (2005). Triphenyltin and tributyltin inhibit pig testicular 17beta-hydroxysteroid dehydrogenase activity and suppress testicular testosterone biosynthesis. *Steroids* 70 645–651. 10.1016/j.steroids.2005.03.005 15899506

[B33] O’Shaughnessy P. J. Monteiro A. Fowler P. A. Morris I. D. (2014). Identification of leydig cell-specific mrna transcripts in the adult rat testis. *Reproduction* 147 671–682. 10.1530/rep-13-0603 24505118

[B34] Purves-Tyson T. D. Handelsman D. J. Double K. L. Owens S. J. Bustamante S. Weickert C. S. (2012). Testosterone regulation of sex steroid-related mrnas and dopamine-related mrnas in adolescent male rat substantia nigra. *BMC Neurosci.* 13:95. 10.1186/1471-2202-13-95 22867132 PMC3467168

[B35] Quinlan A. R. Hall I. M. (2010). Bedtools: a flexible suite of utilities for comparing genomic features. *Bioinformatics* 26 841–842. 10.1093/bioinformatics/btq033 20110278 PMC2832824

[B36] Rapoport S. I. (1988). Brain evolution and alzheimer’s disease. *Rev. Neurol.* 144 79–90. 2898165

[B37] Robinson M. D. McCarthy D. J. Smyth G. K. (2010). Edger: a bioconductor package for differential expression analysis of digital gene expression data. *Bioinformatics* 26 139–140. 10.1093/bioinformatics/btp616 19910308 PMC2796818

[B38] Rommerts F. F. Grootenhuis A. J. Hoogerbrugge J. W. van der Molen H. J. (1985). Ethane dimethane sulphonate (eds) specifically inhibits lh stimulated steroidogenesis in leydig cells isolated from mature rats but not in cells from immature rats. *Mol. Cell. Endocrinol.* 42 105–111. 10.1016/0303-7207(85)90097-8 2998903

[B39] Sharpe R. M. Maddocks S. Kerr J. B. (1990). Cell-cell interactions in the control of spermatogenesis as studied using leydig cell destruction and testosterone replacement. *Am. J. Anat.* 188 3–20. 10.1002/aja.1001880103 2161173

[B40] Shima Y. Matsuzaki S. Miyabayashi K. Otake H. Baba T. Kato S. (2015). Fetal leydig cells persist as an androgen-independent subpopulation in the postnatal testis. *Mol. Endocrinol.* 29, 1581–1593. 10.1210/me.2015-1200 26402718 PMC5414671

[B41] Smith L. B. Walker W. H. (2014). The regulation of spermatogenesis by androgens. *Semin Cell Dev. Biol.* 30 2–13. 10.1016/j.semcdb.2014.02.012 24598768 PMC4043871

[B42] Su D. Zhou Y. Hu S. Guan L. Shi C. Wang Q. (2017). Role of gab1/pi3k/akt signaling high glucose-induced cardiomyocyte apoptosis. *Biomed. Pharmacother.* 93 1197–1204. 10.1016/j.biopha.2017.07.063 28738535

[B43] Taylor M. F. de Boer-Brouwer M. Woolveridge I. Teerds K. J. Morris I. D. (1999). Leydig cell apoptosis after the administration of ethane dimethanesulfonate to the adult male rat is a fas-mediated process. *Endocrinology* 140 3797–3804. 10.1210/endo.140.8.6919 10433241

[B44] Taylor M. F. Woolveridge I. Metcalfe A. D. Streuli C. H. Hickman J. A. Morris I. D. (1998). Leydig cell apoptosis in the rat testes after administration of the cytotoxin ethane dimethanesulphonate: role of the bcl-2 family members. *J. Endocrinol.* 157 317–326. 10.1677/joe.0.1570317 9659295

[B45] Teerds K. J. De Rooij D. G. Rommerts F. F. Wensing C. J. (1988). The regulation of the proliferation and differentiation of rat leydig cell precursor cells after eds administration or daily hcg treatment. *J. Androl.* 9 343–351. 10.1002/j.1939-4640.1988.tb01061.x 2853150

[B46] Trapnell C. Pachter L. Salzberg S. L. (2009). Tophat: discovering splice junctions with RNA-Seq. *Bioinformatics* 25 1105–1111. 10.1093/bioinformatics/btp120 19289445 PMC2672628

[B47] Wang Y. Xie L. Tian E. Li X. Wen Z. Li L. (2019). Oncostatin m inhibits differentiation of rat stem leydig cells *in vivo* and *in vitro*. *J. Cell. Mol. Med.* 23 426–438. 10.1111/jcmm.13946 30320465 PMC6307848

[B48] Wen Q. Cheng C. Y. Liu Y. X. (2016). Development, function and fate of fetal leydig cells. *Semin. Cell Dev. Biol.* 59 89–98. 10.1016/j.semcdb.2016.03.003 26968934 PMC5016207

[B49] Wen Y. Liu G. Zhang Y. Li H. (2019). Microrna-205 is associated with diabetes mellitus-induced erectile dysfunction via down-regulating the androgen receptor. *J. Cell. Mol. Med.* 23 3257–3270. 10.1111/jcmm.14212 30729682 PMC6484320

[B50] Wu X. Liu J. Duan Y. Gao S. Lü Y. Li X. (2017). A short-term exposure to tributyltin blocks leydig cell regeneration in the adult rat testis. *Front. Pharmacol.* 8:704. 10.3389/fphar.2017.00704 29075189 PMC5643909

[B51] Yang Q. Han L. Li J. Xu H. Liu X. Wang X. (2019). Activation of nrf2 by phloretin attenuates palmitic acid-induced endothelial cell oxidative stress via ampk-dependent signaling. *J. Agric. Food Chem.* 67 120–131. 10.1021/acs.jafc.8b05025 30525573

[B52] Ye L. Li X. Li L. Chen H. Ge R. S. (2017). Insights into the development of the adult leydig cell lineage from stem leydig cells. *Front. Physiol.* 8:430. 10.3389/fphys.2017.00430 28701961 PMC5487449

[B53] Yoshikawa Y. Takano O. Kato I. Takahashi Y. Shima F. Kataoka T. (2017). Ras inhibitors display an anti-metastatic effect by downregulation of lysyl oxidase through inhibition of the ras-pi3k-akt-hif-1alpha pathway. *Cancer Lett.* 410 82–91. 10.1016/j.canlet.2017.09.017 28951129

[B54] Yu S. Zhang P. Dong W. Zeng W. Pan C. (2017). Identification of stem leydig cells derived from pig testicular interstitium. *Stem Cells Int.* 2017:2740272. 10.1155/2017/2740272 PMC5294379 28243257

[B55] Zang Z. J. Wang J. Chen Z. Zhang Y. Gao Y. Su Z. (2017). Transplantation of cd51(+) stem leydig cells: a new strategy for the treatment of testosterone deficiency. *Stem Cells* 35 1222–1232. 10.1002/stem.2569 28090714

[B56] Zeng Y. Zhu J. Shen D. Qin H. Lei Z. Li W. (2017). Microrna-205 targets smad4 in non-small cell lung cancer and promotes lung cancer cell growth *in vitro* and *in vivo*. *Oncotarget* 8 30817–30829. 10.18632/oncotarget.10339 28199217 PMC5458170

[B57] Zhang L. Duan H. B. Yang Y. S. (2017). Knockdown of rap2b inhibits the proliferation and invasion in hepatocellular carcinoma cells. *Oncol. Res.* 25 19–27. 10.3727/096504016x14685034103914 28081729 PMC7840814

[B58] Zhang M. Wang J. Deng C. Jiang M. H. Feng X. Xia K. (2017). Transplanted human p75-positive stem leydig cells replace disrupted leydig cells for testosterone production. *Cell Death Dis.* 8:e3123. 10.1038/cddis.2017.531 29022899 PMC5680910

[B59] Zhang P. Chen X. Zheng Y. Zhu J. Qin Y. Lv Y. (2017). Long-term propagation of porcine undifferentiated spermatogonia. *Stem Cells Dev.* 26 1121–1131. 10.1089/scd.2017.0018 28474535 PMC5563923

[B60] Zhang P. Wang J. Lang H. Wang W. Liu X. Liu H. (2018). Microrna-205 affects mouse granulosa cell apoptosis and estradiol synthesis by targeting creb1. *J. Cell. Biochem.* 10.1002/jcb.28133 [Epub ahead of print]. 30556190

[B61] Zirkin B. R. Papadopoulos V. (2018). Leydig cells: formation, function, and regulation. *Biol. Reprod.* 99 101–111. 10.1093/biolre/ioy059 29566165 PMC6044347

