# Supplementary figures and images for: miR-205 Expression Elevated With EDS Treatment and Induced Leydig Cell Apoptosis by Targeting RAP2B via the PI3K/AKT Signaling Pathway
Source: Front Cell Dev Biol. 2020 Jun 9;8:448. doi: 10.3389/fcell.2020.00448 (PMC7300349; doi:10.3389/fcell.2020.00448)

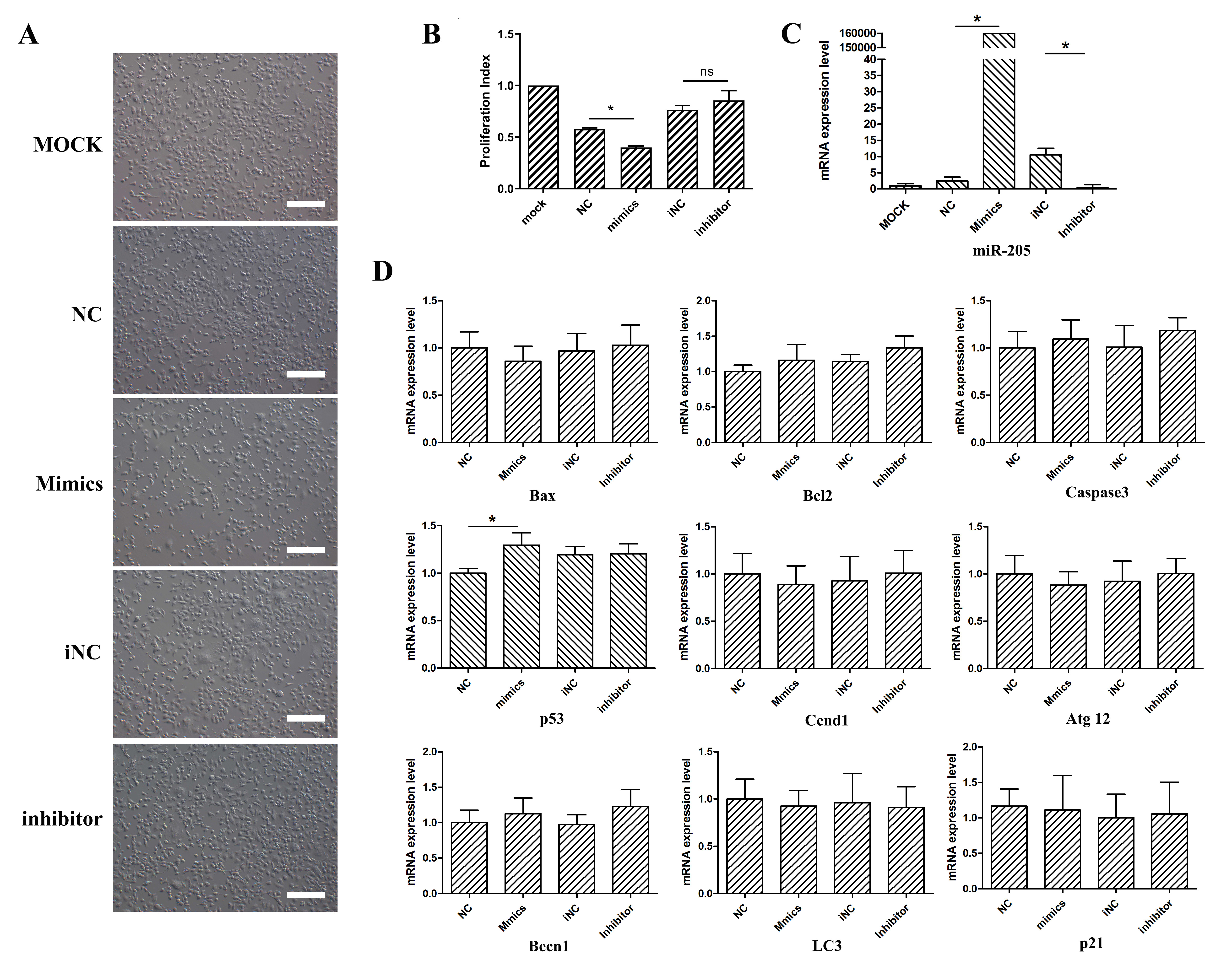

Supplement: FIGURE S1 — The phenotype and several genes expression levels after miR-205 treatment in GC-1spg cells. (A) The phenotype produced by overexpress or knock down miR-205 in GC-1spg cells (bar = 50 μm). (B) Cell viability of GC-1spg cells after miR-205 treatment. (C) miR-205 expression level after overexpress or knock down treatment for 48 h. (D) Apoptosis, proliferation and autophagy related genes expression after miR-205 treatment for 48 h. ∗ represent P < 0.05. ns means not significant. [file Image_1.JPEG]

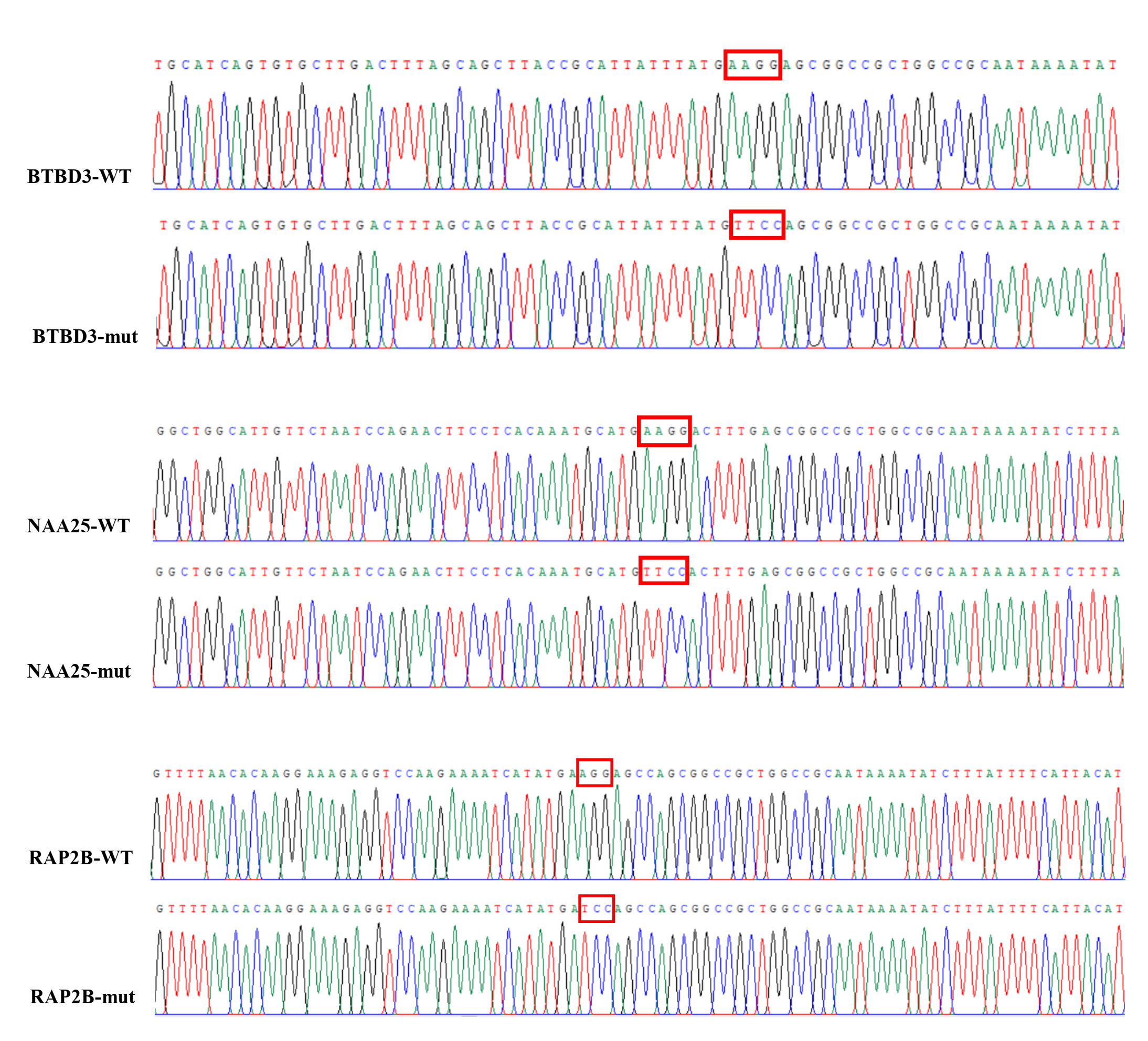

Supplement: FIGURE S4 — The sequencing results of BTBD3, NAA25, and RAP2B dual-luciferase reporter vector constructs. Red box indicates the mutated sequence. [file Image_4.JPEG]
