# Supplementary material for: miR-205 Expression Elevated With EDS Treatment and Induced Leydig Cell Apoptosis by Targeting RAP2B via the PI3K/AKT Signaling Pathway
Source: Front Cell Dev Biol. 2020 Jun 9;8:448. doi: 10.3389/fcell.2020.00448 (PMC7300349; doi:10.3389/fcell.2020.00448)
Supplement: TABLE S1 — Primer sequences for genes designed and used in this study. [file Table_1.DOCX]

**Supplementary table 1** Primer sequences for genes designed and used in this study.

| Primers | Primer sequences (5’-3’) | Length of production/bp |
| --- | --- | --- |
| *INSL3* | F: CAGGAGGCGCCAGAGAAGCTGTGC | 312 |
|  | R: GGGACAGAGGGTCAGCAAGTCTTG |  |
| *PDGFRB* | F: ATGTCACAGTCGTCGAGAGC | 148 |
|  | R: GTCCGGTTGTCCTTGAACCA |  |
| *HSD11B2* | F: TCATCACCGGCTGTGACTC | 101 |
|  | R: AGGGCTATCCAACTCCAACA |  |
| *HSD17B3* | F: GGCTTTTGTGTGCACGTTCT | 236 |
|  | R: TCAGGCTCAGAATGATCGCC |  |
| *NR5A1* | F: CGAGGACCTGGACGAACTG | 236 |
|  | R: GGAAGCGGCAGAAGGGA |  |
| *NR2F1* | F: GGACAAGTCGAGCGGCAA | 228 |
|  | R: GGTTGAGTTGGGGGCATTCT |  |
| *AR* | F: GTACCTGTGTGCCAGCAGA | 238 |
|  | R: GGGCTGACACTCATAGCCTT |  |
| *CYP19A1* | F: GCATCATGCTGGACACCTCT | 101 |
|  | R: AGCTTGCCATGCATCAAAAT |  |
| *CYP26B1* | F: CGCTTGTTCACGCCTGTTTC | 96 |
|  | R: GTACATGACGCTCCAGCCTT |  |
| *HAS1* | F: CTCGGCGACTCGGTGGACTAC | 250 |
|  | R: GGGGACCACTGATGCAGGACA |  |
| *NR4A3* | F: TCTGAGACGTGGTCCATCCA | 210 |
|  | R: CACTGAATGCTCTTGGGGCT |  |
| *CCL4* | F: TTCACATACACCGTGCGGAA | 148 |
|  | R: ACTCCTGGACCCAGTCATCA |  |
| *AMCFII* | F: ATGAGACTCCTAACCAGTCG | 288 |
|  | R: GTCCAGACAGACTTCCTTTC |  |
| *ND1* | F: AGCCACATCCTCAATCTCC | 205 |
|  | R: CCCGATGAGTGCGTATTTT |  |
| *XIRP1* | F: GACACAGCCCCTAGACCAAC | 150 |
|  | R: TGCTCCCGTTGGTGGATTAC |  |
| *HOMER1* | F: TGCGTTTTCCGACACGTAGA | 105 |
|  | R: ACGTTCAAACAGAGGTGGCA |  |
| *P450scc* | F: ACCGTTTCTGGAAGGAGAAGG | 135 |
|  | R: CCACATCTTCAGGGTCGATG |  |
| *PDGFRα* | F: GTGGAGAATCTGCTGCCTGG | 133 |
|  | R: TGTAGGTGACGCCGATGTAG |  |

| Primers | Primer sequences (5’-3’) | Length of production/bp |
| --- | --- | --- |
| *CYP17A1* | F: ATTGACTCCAGCATTGGCGA | 179 |
|  | R: CCGAAGGGCAAGTAGCTCAA |  |
| *StAR* | F: GGTTCTCAGCTGGAAGACACT | 146 |
|  | R: ACCTCGTCCCCATTCTCCTG |  |
| *3β-HSD* | F: TGCAGGAGATCCGGGTACTA | 125 |
|  | R: CTTCAGGCACTGCTCATCCA |  |
| *LHR* | F: GCCTCAGCCGACTATCACTC | 144 |
|  | R: GGAGGTTGTCAAAGGCATTAGC |  |
| *Map1-LC3* | F: CACTGCTCTGTCTTGTGTAGGTTG | 171 |
|  | R: TCGTTGTGCCTTTATTAGTGCATC |  |
| *Atg12* | F: TCCGTGCCATCACATACACA | 242 |
|  | R: TAAGACTGCTGTGGGGCTGA |  |
| *Beclin* | F: TGAATGAGGATGACAGTGAGCA | 248 |
|  | R: CACCTGGTTCTCCACACTCTTG |  |
| *Caspase3* | F: AGCTGGACTGTGGCATTGAG | 143 |
|  | R: CCACGACCCGTCCTTTGAAT |  |
| *Caspase9* | F: ATATCTTCAACGGGAGCGGC | 123 |
|  | R: TGCCTTGAGAGGAAGTGCAG |  |
| *Bax* | F: GCACGTCCACGATCAGTCA | 163 |
|  | R: ACCCTGTAGCAAAAAGGCCC |  |
| *Bcl2* | F: CTTTGAGTTCGGTGGGGTCA | 81 |
|  | R: ATCCACAGGGCGATGTTGTC |  |
| *Ccnd1* | F: CATTCCCTTGACTGCCGAGA | 177 |
|  | R: TTGTTCTCATCCGCCTCTGG |  |
| *PCNA* | F: GAACCTCACCAGCATGTCCA | 221 |
|  | R: ATTCACCCGACGGCATCTTT |  |
| *p53* | F: ATGCGGTTCGGGTCCAAAAT | 154 |
|  | R: CTAAATGGCAGTCGTTCTCTCC |  |
| *p21* | F: CCTGGTGATGTCCGACCTG | 103 |
|  | R: CCATGAGCGCATCGCAATC |  |
| *β-actin* | F: CTCCATCATGAAGTGCGACGT | 114 |
|  | R: GTGATCTCCTTCTGCATCCTGTC |  |
| stem-loop RT-miR-155-5p | GTCGTATCCAGTGCAGGGTCCGAGGTGCACTGGATACGACCCCCTAT | — |
| miR-155-5p-F | TGCGGTTAATGCTAATTGTGATAGG | — |
| stem-loop RT-miR-429 | GTCGTATCCAGTGCAGGGTCCGAGGTGCACTGGATACGACACGGCAT | — |
| miR-429-F | TGCGGTAATACTGTCTGGTAATGCC | — |

| Primers | Primer sequences (5’-3’) | Length of production/bp |
| --- | --- | --- |
| stem-loop RT-miR-215 | GTCGTATCCAGTGCAGGGTCCGAGGTGCACTGGATACGACGTCTGTC | — |
| miR-215-F | TGCGGTTGACCTATGAATTGACAGA | — |
| stem-loop RT-miR-133a | GTCGTATCCAGTGCAGGGTCCGAGGTGCACTGGATACGACCAGCTGG | — |
| miR-133a-F | TGCGGTTGGTCCCCTTCAACCAGCT | — |
| stem-loop RT-miR-9843-3p | GTCGTATCCAGTGCAGGGTCCGAGGTGCACTGGATACGACCCAGAGG | — |
| miR-9843-3p-F | TGCGGTCTGTGAACTAGAAACCTCT | — |
| stem-loop RT-miR-183 | GTCGTATCCAGTGCAGGGTCCGAGGTGCACTGGATACGACCAGTGAA | — |
| miR-183-F | TGCGGTATGGCACTGGTAGAATTCA | — |
| stem-loop RT-miR-370 | GTCGTATCCAGTGCAGGGTCCGAGGTGCACTGGATACGACACCAGGT | — |
| miR-370-F | TGCGGTCCTGCTGGGGTGGAACCTG | — |
| stem-loop RT-miR-128 | GTCGTATCCAGTGCAGGGTCCGAGGTGCACTGGATACGACAAAGAGA | — |
| miR-128-F | TGCGGTCACAGTGAACCGGTCTCTT | — |
| stem-loop RT-miR-142-5p | GTCGTATCCAGTGCAGGGTCCGAGGTGCACTGGATACGACAGTAGTG | — |
| miR-142-5p-F | TGCGGTATAAAGTAGAAAGCACTAC | — |
| stem-loop RT-miR-4332 | GTCGTATCCAGTGCAGGGTCCGAGGTGCACTGGATACGACGGCGCCC | — |
| miR-4332-F | TGCGGTACGGCCGCCGCCGGGCGCC | — |
| stem-loop RT-miR-615 | GTCGTATCCAGTGCAGGGTCCGAGGTGCACTGGATACGACAGAGGGA | — |
| miR-615-F | TGCGGTCCGAGCCTGGGTCTCCCTC | — |
| stem-loop RT-miR-194b | GTCGTATCCAGTGCAGGGTCCGAGGTGCACTGGATACGACTCCACAT | — |
| miR-194b-F | TGCGGTGTAACAGCGACTCCATGTG | — |

| Primers | Primer sequences (5’-3’) | Length of production/bp |
| --- | --- | --- |
| stem-loop RT-miR-144 | GTCGTATCCAGTGCAGGGTCCGAGGTGCACTGGATACGACGTACATC | — |
| miR-144-F | TGCGGTACAGTATAGATGATGATGT | — |
| stem-loop RT-miR-21 | GTCGTATCCAGTGCAGGGTCCGAGGTGCACTGGATACGACTCAACAT | — |
| miR-21-F | TGCGGTAGCTTATCAGACTGATGTT | — |
| stem-loop RT-miR-205 | GTCGTATCCAGTGCAGGGTCCGAGGTATTCGCACTGGATACGACCAGACTCC | — |
| miR-205-F | CTGGAGTCCTTCATTCCACCGG | — |
| Reverse Primer | GTGCAGGGTCCGAGGT | — |
| U6 | F: CGCTTCACGAATTTGCGTGTCAT | 107 |
|  | R: GCTTCGGCAGCACATATACTAAAAT |  |
