# Supplementary material for: miR-205 Expression Elevated With EDS Treatment and Induced Leydig Cell Apoptosis by Targeting RAP2B via the PI3K/AKT Signaling Pathway
Source: Front Cell Dev Biol. 2020 Jun 9;8:448. doi: 10.3389/fcell.2020.00448 (PMC7300349; doi:10.3389/fcell.2020.00448)
Supplement: TABLE S2 — Primers for amplify 3′-UTR of miR-205 target genes. [file Table_2.DOCX]

**Supplementary table 2** Primers for amplify 3’-UTR of miR-205 target genes

| Predicted target genes | The sequence of primers (5'-3') | Length of production/bp |
| --- | --- | --- |
| *SRSF10* | F: CCGCTCGAGTTTAGTCCTTGCTTGTAGAC | 457 |
|  | R: ATTTGCGGCCGCCATTATTTCCTTCATTTTGT |  |
|  | mutR: ATTTGCGGCCGCCATTATTTGGAACATTTTGT |  |
| *RAP2B* | F: CCGCTCGAGACTTGTGTAGCCAGCATCCC | 342 |
|  | R: ATTTGCGGCCGCTGGCTCCTTCATATGATTTTCTTGG |  |
|  | mutR: ATTTGCGGCCGCTGGCTGGATCATATGATTTTCTTGG |  |
| *BTBD3* | F: CCGCTCGAGTGCAGAGTGACTAGAAGGCA | 755 |
|  | R: ATTTGCGGCCGCTCCTTCATAAATAATGCGGTAAGC |  |
|  | mutR: ATTTGCGGCCGCTGGAACATAAATAATGCGGTAAGC |  |
| *SLC35B3* | F: CCGCTCGAGTGGGAACAACCATTAATCAATTCAC | 204 |
|  | R: ATTTGCGGCCGCGCACTTTCCTTCATACTGACTTT |  |
|  | mutR: ATTTGCGGCCGCGCACTTTGGAACATACTGACTTT |  |
| *HS3ST1* | F: CCGCTCGAGCTCACCGTGGAGAAGACACC | 852 |
|  | R: ATTTGCGGCCGCCAGTGCAATAGTTAAGTTCCTTCA |  |
|  | mutR: ATTTGCGGCCGCCAGTGCAATAGTTAAGTTGGAACA |  |
| *CADM1* | F: CCGCTCGAGTCGACTCTGTTCTTTGAAAGCG | 827 |
|  | R: ATTTGCGGCCGCTTTGGCATCAATTATACATCCTTCA |  |
|  | mutR: ATTTGCGGCCGCTTTGGCATCAATTATACATGGAACA |  |
| *NAA25* | F: CCGCTCGAGGATGATGTGCAAGCAGCGAGG | 592 |
|  | R: ATTTGCGGCCGCTCAAAGTCCTTCATGCATTTGTGAG |  |
|  | mutR: TTTGCGGCCGCTCAAAGTGGAACATGCATTTGTGAG |  |
